# Effect of chemokine receptor CXCR4 on hypoxia-induced pulmonary hypertension and vascular remodeling in rats

**DOI:** 10.1186/1465-9921-12-21

**Published:** 2011-02-04

**Authors:** Lunyin Yu, Charles A Hales

**Affiliations:** 1 Pulmonary and Critical Care Unit, Department of Medicine, Massachusetts General Hospital, Harvard Medical School, Boston, MA 02114, USA

## Abstract

**Background:**

CXCR4 is the receptor for chemokine CXCL12 and reportedly plays an important role in systemic vascular repair and remodeling, but the role of CXCR4 in development of pulmonary hypertension and vascular remodeling has not been fully understood.

**Methods:**

In this study we investigated the role of CXCR4 in the development of pulmonary hypertension and vascular remodeling by using a CXCR4 inhibitor AMD3100 and by electroporation of CXCR4 shRNA into bone marrow cells and then transplantation of the bone marrow cells into rats.

**Results:**

We found that the CXCR4 inhibitor significantly decreased chronic hypoxia-induced pulmonary hypertension and vascular remodeling in rats and, most importantly, we found that the rats that were transplanted with the bone marrow cells electroporated with CXCR4 shRNA had significantly lower mean pulmonary pressure (mPAP), ratio of right ventricular weight to left ventricular plus septal weight (RV/(LV+S)) and wall thickness of pulmonary artery induced by chronic hypoxia as compared with control rats.

**Conclusions:**

The hypothesis that CXCR4 is critical in hypoxic pulmonary hypertension in rats has been demonstrated. The present study not only has shown an inhibitory effect caused by systemic inhibition of CXCR4 activity on pulmonary hypertension, but more importantly also has revealed that specific inhibition of the CXCR4 in bone marrow cells can reduce pulmonary hypertension and vascular remodeling via decreasing bone marrow derived cell recruitment to the lung in hypoxia. This study suggests a novel therapeutic approach for pulmonary hypertension by inhibiting bone marrow derived cell recruitment.

## Introduction

Pulmonary hypertension caused by many chronic lung diseases associated with prolonged hypoxia can result in right ventricular hypertrophy and heart failure. Although available treatments can improve prognosis, this disease has been incurable with poor survival. An important pathological feature of pulmonary hypertension is increased medial thickening of pulmonary artery resulting from hypertrophy and hyperplasia of the pulmonary artery smooth muscle cells (PASMC) [1-3].

The CXC chemokine receptor 4(CXCR4) is the receptor for CXCL12, one of chemokines. Chemokines are a family of small cytokines or proteins secreted by cells, which have the ability to induce directed chemotaxis in nearby responsive cells and therefore are also called chemotactic cytokines. Chemokines include at least 40 ligands and 20 receptors [4]. According to amino acid motif in their N-termini, chemokine ligands can be categorized into four types, C, CC, CXC and CX_3_C. The CXC chemokines contain two N-terminal cysteins separated by one amino acid, thus represented in its name with an "X" [5,6]. CXCR4 is one of the seven CXC motif chemokine receptors found so far.

The interaction of CXCR4 and its unique ligand CXCL12 is essential for migration of progenitor cells during embryonic development of the cardiovascular, hemopoietic and central nervous system. CXCR4 is also involved in vascular remodeling [7-9]. Nemenoff and colleagues reported that the CXCL12/CXCR4 axis is involved in vascular remodeling and recruitment of progenitor cells [10]. Karshovska and co-workers found that neointima formation and smooth muscle progenitor cell mobilization were inhibited by CXCR4 inhibitor after arterial injury [11]. Zernecke et al. found that the CXCL12/CXCR4 axis played an important role in neointimal hyperplasia and recruitment of smooth muscle progenitor cells after arterial injury [12]. Satoh and colleagues [13] observed that pravastatin attenuated hypoxic pulmonary hypertension was accompanied by a decrease in plasma level of CXCL12 and in accumulation of CXCR4^+ ^cells in mouse lungs.

The CXCL12/CXCR4 axis was originally described as a regulator of cell interaction in the immune system [14] mediating leukocyte migration to inflammatory area [15]. This axis was also involved in regulation of wide range of cell migration or mobilization [16-19]. In addition, it has been reported that CXCR4 plays a vital role in regulation of stem/progenitor cell migration and development in cancer, nervous system and heart repair after myocardial infarction [20-25]. Young et al. [26] recently used a neonatal mouse model of pulmonary hypertension and found that the inhibition of CXCR4 activity significantly decreased hypoxia-induced pulmonary hypertension. Interestingly, Gambaryan et al. most recently reported that AMD3100, an antagonist of CXCR4, prevented in part pulmonary hypertension, vascular remodeling and right ventricular hypertrophy induced by chronic hypoxia in mice [27]. However, the role of CXCR4 in pulmonary hypertension and remodeling has not been completely understood.

In this study we used a CXCR4 inhibitor, AMD3100, in rats to determine the role of CXCR4 in development of pulmonary hypertension and vascular remodeling. In addition, we electroporated CXCR4 shRNA into bone marrow cells and then transplanted the bone marrow cells with CXCR4 shRNA into rats to investigate the effect of CXCR4 on bone marrow cell migration in hypoxia-induced pulmonary hypertension. We hypothesized that inhibition of systemic CXCR4 through administration of AMD3100 will inhibit hypoxia-induced pulmonary hypertension and vascular remodeling in rats and that specific inhibition of the CXCR4 in bone marrow cells also will impact development of pulmonary hypertension and vascular remodeling induced by chronic hypoxia.

## Materials and methods

### Chemicals

AMD3100 octahydrochloride hydrate (AMD3100) (1,1'-[1,4-Phenylenebis(methylene)]bis-1,4,8,11-tetraazacyclotetradecane octahydrochloride) was obtained from Sigma. CXCR4 shRNA plasmid, a plasmid vector containing the shRNA under control of the U1 promoter, was obtained from SABiosciences (Frederick, MD).

### Animals

Animal experiments were approved by the Subcommittee on Research Animal Care at Massachusetts General Hospital. Wild type male Sprague-Dawley (SD) rats (Charles River Laboratories, Wilmington, MA), weighing 150 ~ 200 grams, were used as bone marrow cell transplant recipients. Male SD background transgenic rats containing green fluorescent protein gene (SD-Tg(GFP)2BalRrrc, termed as SD-GFP) were obtained from Resource and Research Center at University of Missouri (Columbia, MO) and used as bone marrow cell donors.

### CXCR4 inhibitor and hypoxic pulmonary hypertension

Rats were placed in a hypoxia chamber and treated with a CXCR4 inhibitor AMD3100. The CXCR4 inhibitor was administered by a mini osmotic pump (DURECT Corporation, Cupertine, CA) implanted subcutaneously at dose of 10 mg/kg/day for 14 days. The control animals received normal saline by the same size mini pump. After two weeks of exposure to hypoxia and treatment with the CXCR4 inhibitor, the rats were removed from hypoxia for measurements.

### Electroporation of bone marrow cells with CXCR4 shRNA and hypoxic pulmonary hypertension

This experiment included bone marrow cell harvest, CXCR4 shRNA electroporation, transplantation and then pulmonary hypertension development. Bone marrow cells were harvested from donor SD-GFP rats following the methods described by Spees [28] and Kroeger [29]. Briefly, SD-GFP rats were sacrificed by CO_2 _exposure and femurs and tibias of the rats were dissected sterilely. After cutting each end of the femurs and tibias to expose marrow, we placed each bone into a 1.5 ml sterile eppendorf tube and centrifuged it for 1 min at 1200 rpm. Bone marrow pellets were obtained and resuspended with PBS and then filtered through 70 micro cell strainers. Followed by centrifugation, the bone marrow cells were resuspended with medium and the number of the bone marrow cells was counted for transplantation. Electroporation of CXCR4 shRNA plasmid into bone marrow cells was performed following published methods [30-33]. Briefly, the harvested bone marrow cells (5 × 10^6 ^cells per rat) were resuspended with serum free medium at 1 × 10^6 ^cells/ml and then placed into an electroporation cuvette. After adding CXCR4 shRNA plasmid (2 μM) to the cuvette and placing the cuvette in an electroporator chamber (Bio-Rad, GenePulser Xcell), the cells were then electroporated following the manufacturer's instruction. After electroporation, the cell suspension was transfered to a centrifuge tube, spun down and resuspended with medium for transplantation. The efficiency of the shRNA delivery was detected by Western blot. To allow transplantation of the bone marrow cells, SD receipt rats were lethally irradiated with a dose of 11 Gy. Following irradiation, the harvested bone marrow cells were injected into the rat via tail vein (5 × 10^6 ^cells per rat). After transplantation, the rats were recovered in normoxia for 3 weeks before exposure to hypoxia.

### Hypoxia exposure

Hypoxia exposure was performed as previously described [34-37]. Briefly, animals were weighed and placed in a tightly sealed hypoxia chamber or exposed to normoxia for two weeks. Oxygen concentration was maintained at 10% by controlling the flow rates of compressed air and N_2_. Concentrations of O_2 _and CO_2 _in chamber were checked daily.

### Measurement of mean pulmonary artery pressure

The measurement for mean pulmonary artery pressure (mPAP) was performed as described previously [34-37]. Briefly, after 14 days in the chamber the animals were removed and anesthetized with intraperitoneal ketamine (80 mg/kg) and diazepam (5 mg/kg). Animals were placed on a warming blanket to maintain body temperature at 37°C. mPAP was measured via a catheter (0.012" × 0.021" silicone tubing) passed through the right external jugular vein and right ventricle. Once the mPAP was obtained, the animals were sacrificed with 200 mg/kg of pentobarbital and used immediately for the determination of right ventricular hypertrophy, hematocrit, and lung pathology.

### Measurement of right ventricular hypertrophy

The ventricles and septum of the animals were collected and the wet and dry ventricle and septal weight were obtained by drying them for 24 hours at 60°C. Then a ratio of right ventricle to left ventricle plus septum weight (RV/(LV+S)) was calculated for determination of right ventricular hypertrophy [34-37].

### Measurement of pulmonary vascular remodeling

Elastic fibers in pulmonary arteries were stained for measurement of medial wall thickness of pulmonary arteries. Percent medial wall thickness of pulmonary arteries was used for evaluation of pulmonary artery remodeling as previously described [36,37]. The percent wall thickness was calculated as average diameter of the external elastic lamina minus the average diameter of internal elastic lamina divided by the average diameter of external elastic lamina. A computer imaging analysis was applied for measurement of wall thickness. Images of individual pulmonary arteries were captured using a digital camera, mounted on a light microscope and linked to a computer. All the muscular arteries between 50 μm and 150 μm in diameter in slides were analyzed in this study. The detail on measurement of wall thickness had been described previously [35,36].

### Hematocrit analysis

Blood samples were centrifuged in microcapillary tubes for 3 min and the hematocrit was read directly.

### Western blot

Total protein was isolated from rat bone marrow cells, rat lungs and pulmonary arteries isolated from rats that received bone marrow cell transplantation. Western blot was performed as described previously [34,35,38,39]. Antibodies included CXCR4 (Abcam, Cambridge, MA), c-kit (Santa Cruz Biotechnology, Inc., Santa Cruz, CA), GFP and GAPDH (Abcam, Cambridge, MA).

### Analysis of bone marrow cell engraftment

Bone marrow white blood cell (WBC) count and flow cytometry were performed for this analysis. The WBC numbers were determined by directly counting WBC number in bone marrow under the microscope by using a hemacytometer after staining the bone marrow cells with crystal violet. For flow cytometry analysis, bone marrow mononuclear cells were collected by using density gradient centrifugation media (Ficoll-Paque Premium, GE Healthcare Bio-Sciences AB, Uppsala, Sweden). Mononuclear cells were stained with primary antibodies, anti-mouse/rat CD34 (R&D Systems, Inc. Minneapolis, MN) and anti-rat CD45 (BioLegend, San Diego, CA). Following incubation for 30 minutes and washing with PBS, the cells were incubated with secondary antibody for 30 minutes and then flow cytometric analysis was performed with a 7 Laser SORP BD LSR II. Data were collected with DIVA software on LSR II and analyzed with FlowJo v8.8.6.

### Statistical Analysis

Statistics was performed using the computer program Statview (SAS Institute Inc., Cary, NC) with the analysis of variance (ANOVA). If ANOVA was significant, multiple comparisons were made among groups using the Fisher protected least significant difference test. All values were expressed as the mean ± standard error. Significance was set at p < 0.05.

## Results

### Administration of CXCR4 inhibitor significantly decreased hypoxia-induced pulmonary artery pressure and right ventricular hypertrophy in rats

**Figure 1:**
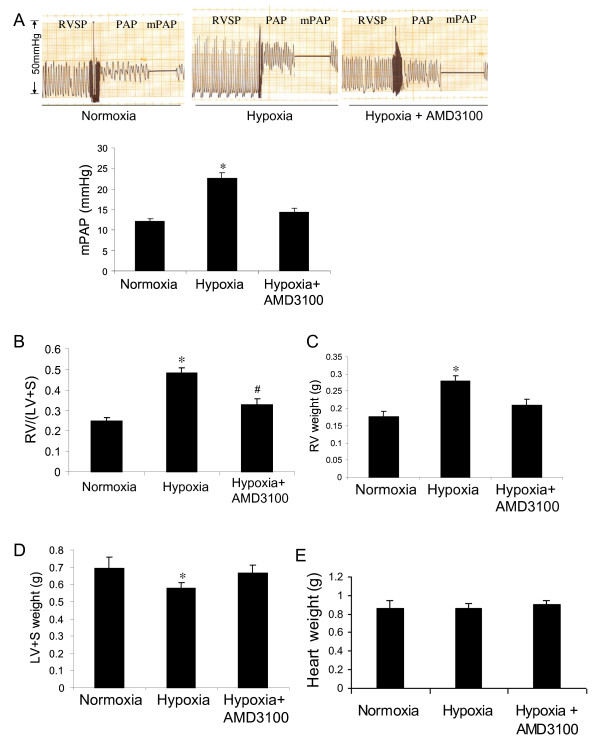
**Effect of CXCR4 inhibitor on pulmonary artery pressure and right ventricular hypertrophy induced by chronic hypoxia in rats**. (A) mPAP, showing representative tracings of pulmonary artery pressure (upper panel) and quantitative data (lower panel). (B-E) Right ventricular hypertrophy, showing data on RV/(LV+S) (B), right ventricular weight (C), left ventricular plus septal weight (D) and whole heart weight (E). *p < 0.05 as compared with other groups and # p > 0.05 as compared with normoxic control rats. n = 5 rats for each group.

After two weeks of exposure to hypoxia, control rats developed pulmonary hypertension, showing a significant increase in pulmonary artery pressure (mPAP) as compared with the normoxic rats. However, the pulmonary artery pressure was significantly decreased in the animals treated with the CXCR4 inhibitor as compared with the hypoxia controls (Figure 1A). The CXCR4 inhibitor also significantly decreased right ventricular hypertrophy, showing a decrease in the ratio of RV/(LV+S) in the rats treated with the CXCR4 inhibitor as compared with the hypoxic control animals (Figure 1B). Interestingly, we found that exposure to hypoxia significantly increased right ventricular weight (Figure 1C) and decreased left ventricular plus septal weight (Figure 1D), which resulted in an increase in the ratio of RV/(LV+S) in hypoxic control animals, but the whole heart weight was not different between the hypoxic control and hypoxia plus CXCR4 inhibitor treatment (Figure 1E).

### Administration of CXCR4 inhibitor significantly decreased hypoxia-induced pulmonary artery remodeling in rats

**Figure 2:**
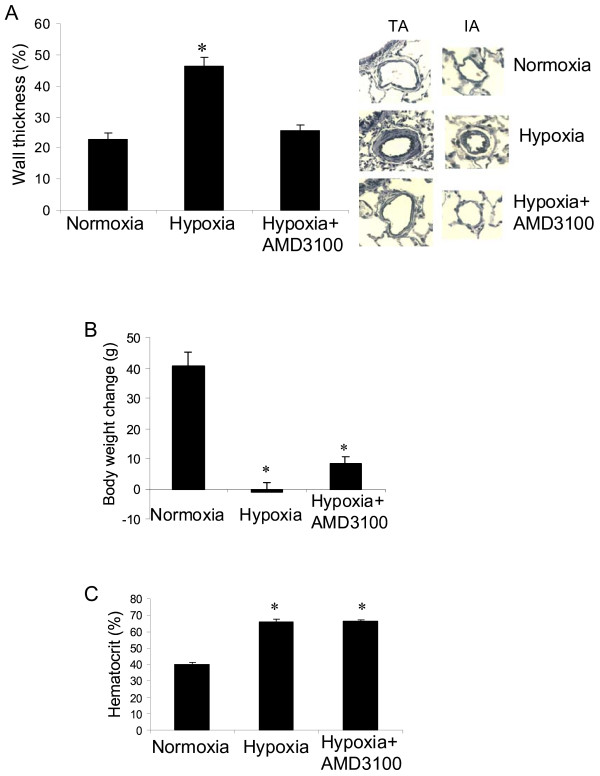
**Effect of CXCR4 inhibitor on wall thickness of pulmonary arteries induced by chronic hypoxia in rats**. (A) Wall thickness showing quantitative data on percent wall thickness (%WT) (left panel) and representative microphotographs (right panel). TA = terminal bronchial arterioles; I A = intra-acinous arterioles. (B) Body weight change. *p < 0.05 as compared with other groups. (C) hematocrit. *p < 0.05 as compared with normoxia. n = 5 rats for each group.

Exposure to hypoxia significantly induced vascular remodeling, showing an increase in medial wall thickness of pulmonary arteries in hypoxic control group as compared with the normoxic controls. Treatment of rats with the CXCR4 inhibitor significantly prevented the wall thickness of pulmonary arteries induced by hypoxia (Figure 2A). Interestingly, administration of the CXCR4 inhibitor significantly attenuated body weight loss in animals under hypoxia as compared with the hypoxic control rats (Figure 2B). In addition, hypoxia significantly increased hematocrit values in all rats as compared with their normoxic controls, but no significant difference was observed between the hypoxic groups (Figure 2C).

### Electroporation of bone marrow cells with CXCR4 shRNA significantly decreased hypoxia-induced pulmonary hypertension and right ventricular hypertrophy in rats

**Figure 3:**
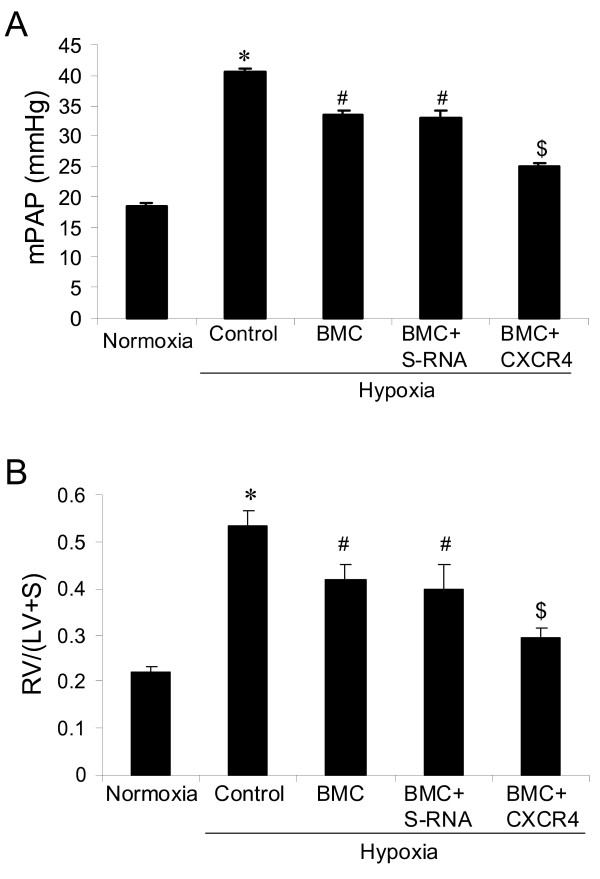
**Effect of electroporation of bone marrow cells with CXCR4 shRNA on hypoxia-induced pulmonary hypertension and right ventricular hypertrophy in rats**: (A) mPAP and (B) RV/(LV+S). *p < 0.05 as compared with other groups; #p < 0.05 as compared with normoxia and BMC+CXCR4; $p < 0.05 as compared with normoxia. n = 5 rats for each group. BMC = transplantation of bone marrow cells without shRNA, BMC+S-RNA = transplantation of bone marrow cells with scrambled shRNA, BMC+ CXCR4 = transplantation of bone marrow cells with CXCR4 shRNA.

After transplantation with bone marrow cells electroporated with CXCR4 shRNA and recovery under normoxia for three weeks (all rats that did not receive bone marrow cell transplantation died within one week after irradiation), the rats were placed in the hypoxia chamber for two weeks to induce pulmonary hypertension. We found that hypoxia-induced pulmonary hypertension was significantly decreased in the rats transplanted with CXCR4 shRNA bone marrow cells, showing decreased mean pulmonary artery pressure (Figure 3A) and decreased ratio of RV/(LV+S) (Figure 3B) as compared with the rats receiving scrambled shRNA in bone marrow cells or the rats injected with bone marrow cells without shRNA.

### Electroporation of bone marrow cells with CXCR4 shRNA significantly decreased hypoxia-induced vascular remodeling

**Figure 4:**
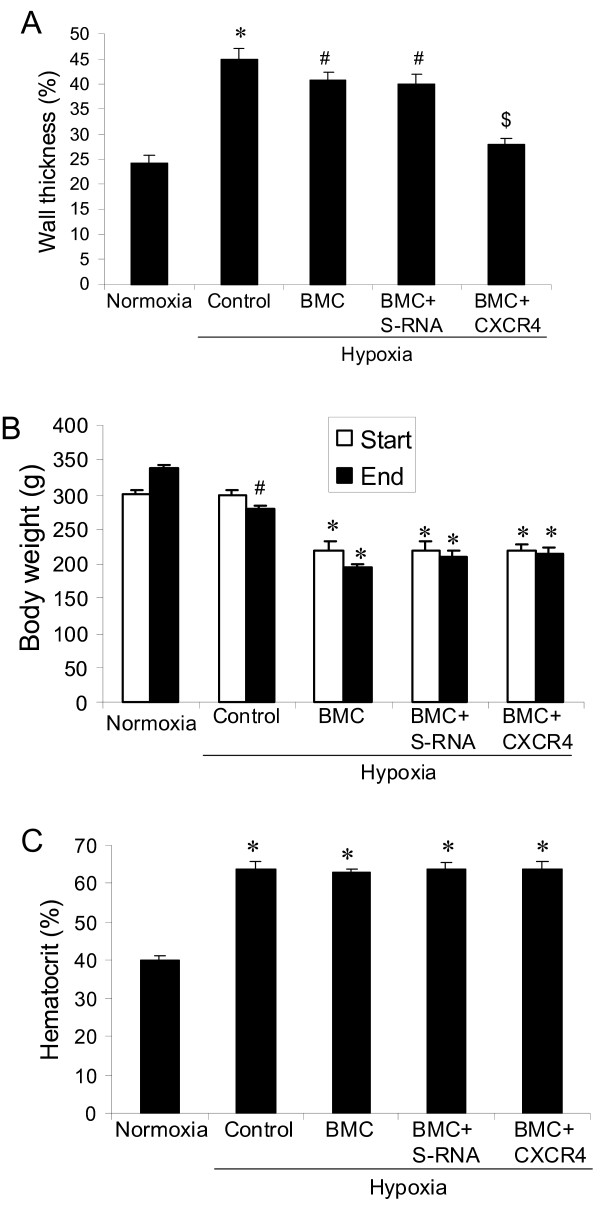
**Effect of electroporation of bone marrow cells with CXCR4 shRNA on hypoxia-induced pulmonary hypertension and vascular remodeling in rats**: (A) Percent wall thickness. *p < 0.05 as compared with normoxia and hypoxic controls. # p < 0.05 as compared with normoxia and BMC+CXCR4. $p < 0.05 as compared with normoxia. (B) Body weight change. # p < 0.05 as compared with normoxia. *p < 0.05 as compared with normoxia and hypoxic controls. (C) hematocrit. *p < 0.05 as compared with normoxia control. n = 5 rats for each group. BMC = transplantation of bone marrow cells without shRNA, BMC+S-RNA = transplantation of bone marrow cells with scrambled shRNA, BMC+ CXCR4 = transplantation of bone marrow cells with CXCR4 shRNA.

We found that transplantation with bone marrow cells electroporated with CXCR4 shRNA significantly decreased hypoxia-induced vascular remodeling, showing a decrease in percent wall thickness of pulmonary arteries (Figure 4A) as compared with other hypoxic control groups. In addition, we found that all animals with bone marrow transplantation had decreased body weight as compared with the rats without bone marrow transplantation (Figure 4B). Interestingly, as shown in the figures (Figure 3A &3B and 4A), the irradiated rats developed lower pulmonary hypertension as compared with non-irradiated hypoxic animals, but there was no significant difference between them. Hypoxia also significantly increased hematocrit values in all hypoxic animals (Figure 4C).

### Effect of CXCR4 shRNA delivery on CXCR4 expression in bone marrow cells

**Figure 5:**
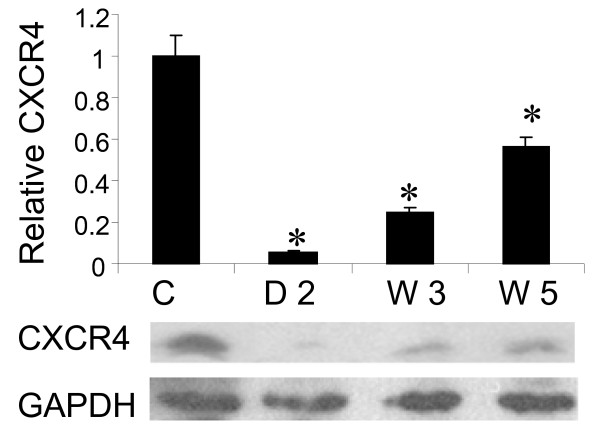
**Effect of CXCR4 shRNA delivery on CXCR4 expression in bone marrow cells**: Western blot on proteins isolated from rat bone marrow cells was performed to analyze CXCR4 expression. Quantitative data (upper panel) and representative images (lower panel). C = control, D2 = day 2, W3 = week 3 and W5 = week 5. *p < 0.05 as compared with control. n = 3 for each groups.

To determine the efficiency of the CXCR4 shRNA delivery in bone marrow cells, we measured CXCR4 expression in primary bone marrow cells and bone marrow cells harvested from recipient rats. Following electroporation of bone marrow cells with CXCR4 shRNA plasmid, we transplanted the bone marrow cells into rats and, at the same time, left some cells and cultured them for 48 hours for analysis of CXCR4 expression in primary bone marrow cells. In addition, we harvested bone marrow cells from the rats that received bone marrow cell transplantation at end of recovery (week 3) and at end of hypoxia exposure (week 5) respectively and measured CXCR4 expression. We found more than 90% inhibition at 48 hours after electroporation in primary bone marrow cells, more than 70% inhibition on week 3 and more than 40% inhibition on week 5 in the harvested bone marrow cells with CXCR4 shRNA electroporation from the recipient rats (Figure 5).

### Effect of CXCR4 shRNA delivery on bone marrow-derived progenitor cell migration

**Figure 6:**
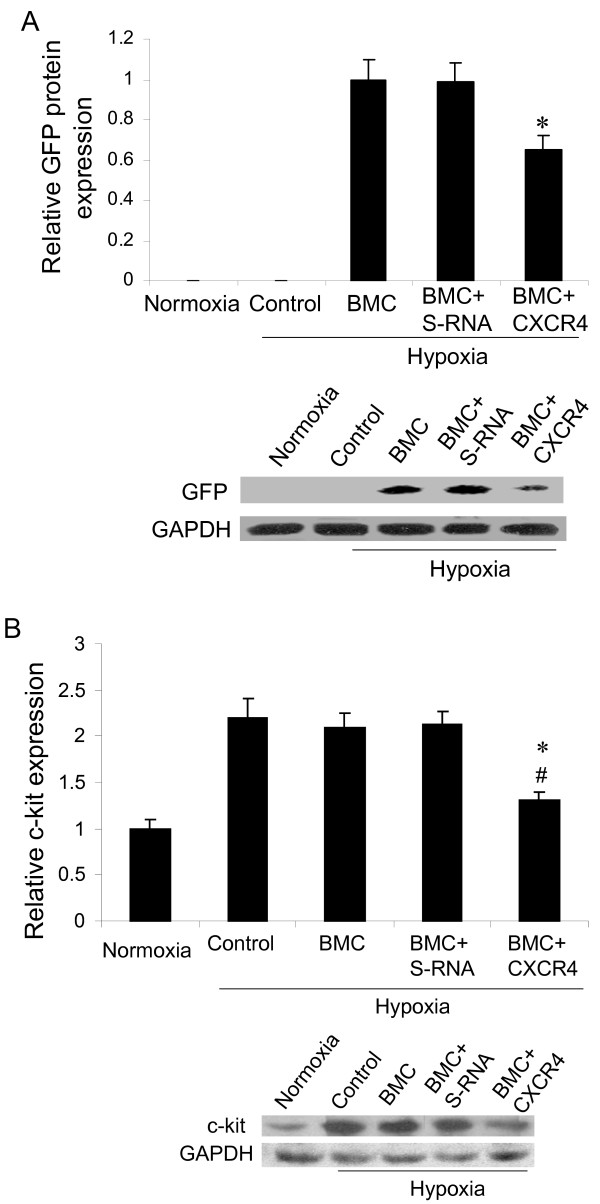
**Effect of CXCR4 shRNA delivery on bone marrow cell migration to rat lung**: (A) GFP expression. Proteins were isolated form rat lungs and Western blot was performed for analysis of GFP protein expression. Quantitative data (upper panel), setting hypoxia BMC as 1, and representative images (lower panel). *p < 0.05 as compared with other groups. (B) c-kit expression. Proteins were isolated form rat pulmonary artery and Western blot was performed for analysis of c-kit expression. Quantitative data (upper panel), setting normoxia control as 1, and representative images (lower panel). *p < 0.05 as compared with other hypoxia groups. # p > 0.05 as compared with normoxia control. n = 3 for each groups. BMC = transplantation of bone marrow cells without shRNA, BMC+S-RNA = transplantation of bone marrow cells with scrambled shRNA, BMC+ CXCR shRNA = transplantation of bone marrow cells with CXCR4 shRNA.

To demonstrate the effect of CXCR4 shRNA delivery on bone marrow cell migration, we detected GFP, a marker for donor bone marrow cells, expression in rat lung. We found a significant decrease in GFP protein expression in the lungs from rats that received CXCR4 shRNA bone marrow cells (Figure 6A) as compared with other hypoxic animals. In order to further determine whether CXCR4 inhibition in bone marrow cells affected bone marrow-derived progenitor cell migration, we measured c-kit, a hematopoietic progenitor marker, expression in pulmonary artery isolated from rats that received bone marrow cells. We found a significant decrease in c-kit expression in the pulmonary artery from the rats that received the bone marrow cells electroporated with CXCR4 shRNA as compared with other hypoxic control groups (Figure 6B).

### Effect of CXCR4 shRNA delivery on engraftment of bone marrow cells

To investigate the effect of CXCR4 shRNA delivery on bone marrow cell engraftment. We measured white blood cells (WBC) in harvested bone marrow cells by counting the number of WBC and analyzed expression of CD34 and CD45 in bone marrow cells by flow cytometry. We found that delivery of CXCR4 shRNA decreased the bone marrow cell engraftment in this study (Table 1), although the change was not significant.

**Table 1:** Effect of CXCR4 shRNA on bone marrow cell engraftment

			Hypoxia		
	Normoxia				
	Control	Control	BMC	BMC/S-R	BMC/CXCR4
WBC(x10^6^)	22.5 ± 1.5	24.6 ± 2.2	24.5 ± 2.4	23.2 ± 2.0	21.3 ± 2.1 ∆
CD34^+^(%)	38.5 ± 2.4	39.1 ± 2.0	38.7 ± 1.9	38.2 ± 3.4	35.1 ± 3.0 ∆
CD45^+^(%)	32.2 ± 1.8	31.4 ± 3.4	32.3 ± 1.3	31.4 ± 3.1	30.0 ± 2.1 ∆

∆p > 0.05 as compared with other groups. n = 3 for each group.

## Discussion

In this study we found that a CXCR4 inhibitor significantly inhibited hypoxia-induced pulmonary hypertension (Figure 1A), right ventricular hypertrophy (Figure 1B) and vascular remodeling of pulmonary arteries (Figure 2A) in rats. We also found that inhibition of the CXCR4 in bone marrow cells by shRNA electroporation also significantly attenuated hypoxia-induced pulmonary hypertension (Figure 3A), right ventricular hypertrophy (Figure 3B) and vascular remodeling (Figure 4A). The delivery of CXCR4 shRNA by electroporation significantly inhibited CXCR4 expression in bone marrow cells at 48 hours and on week 3 and week 5 (Figure 5) and also significantly decreased GFP expression in rat lungs (Figure 6A) and decreased c-kit expression in rat pulmonary artery (Figure 6B).

Recently we found that CXCR4 was expressed in pulmonary artery smooth muscle cells and that hypoxia increased CXCR4 expression in the lungs from mice with pulmonary hypertension and that a CXCR4 inhibitor AMD3100 significantly inhibited pulmonary artery smooth muscle cell proliferation (unpublished data). We thereafter investigated the effect of the CXCR4 inhibitor on hypoxia-induced pulmonary hypertension in rats in this study. As shown in the results, two weeks of treatment with the CXCR4 inhibitor significantly decreased hypoxia-induced pulmonary pressure, right ventricular hypertrophy and vascular remodeling of pulmonary arteries in rats. These results demonstrated that CXCR4 plays a critical role in development of pulmonary hypertension and vascular remodeling in rats. Toshner et al. recently reported up-regulated CXCL12 and CXCR4 in lung tissue from patients with idiopathic pulmonary hypertension [40]. Young et al. [26] and Gambaryan et al. [27] recently reported that inhibition of CXCR4 activity significantly decreased hypoxia-induced pulmonary hypertension in mice, but Young et al. only used neonatal mice [26]. The results from our study further demonstrated the effect of CXCR4 in development of pulmonary hypertension and vascular remodeling in chronically hypoxic rats.

An important pathological feature of pulmonary hypertension is vascular remodeling of the pulmonary arteries. One of the unsolved questions regarding the vascular remodeling of pulmonary arterioles in pulmonary hypertension is whether the vascular remodeling is caused by bone marrow-derived progenitor cells, which migrate to the wall of pulmonary arteries via bloodstream [1]. Although some work has been done on bone marrow stem cells and pulmonary hypertension in different laboratories [28,41,42], the results were not consistent. We in this study investigated relationship between bone marrow cell migration and development of pulmonary hypertension. We electroporated CXCR4 shRNA into bone marrow cells to inhibit CXCR4 and then transplanted the bone marrow cells into lethally irradiated rats. After two weeks of exposure to hypoxia, the rats transplanted with CXCR4 shRNA bone marrow cells had significantly lower pulmonary artery pressure, right ventricular hypertrophy and wall thickness of pulmonary arteries as compared with hypoxic control animals that received scrambled shRNA in bone marrow cells or were injected with bone marrow cells without shRNA. Because electroporation of bone marrow cells with the CXCR4 shRNA only affected CXCR4 expression in bone marrow cells, this finding provided direct evidence that CXCR4 is involved in regulation of bone marrow cell migration during development of pulmonary hypertension and vascular remodeling induced by hypoxia. This finding also demonstrated the involvement of bone marrow cells in pulmonary hypertension and vascular remodeling. Although Young et al. reported that inhibition of CXCR4 activity by AMD3100 decreased hypoxia-induced pulmonary hypertension and vascular remodeling in neonatal mice, which was accompanied with decreased expression of some stem cell markers in the mouse lungs, they did not show any direct evidence to demonstrate the relationship between bone marrow cell migration and the development of pulmonary hypertension. Therefore, this is the first study to show that migration inhibition of bone marrow cells by CXCR4 shRNA inhibits development of hypoxia-induced pulmonary hypertension and vascular remodeling. Electroporation is simple and reliable method for delivery of specific gene into primary bone marrow cells [30-33]. Therefore, electroporation of bone marrow cells with specific genes would be a useful method for investigation of bone marrow cells and pulmonary hypertension.

It has been reported that CXCL12/CXCR4 axis plays an important role in cell recruitment [7-9], including mobilization of bone marrow cells [43-45]. In this study, we observed that inhibition of the CXCR4 in bone marrow cells significantly decreased hypoxia-induced pulmonary hypertension and vascular remodeling, which indicated that bone marrow cell migration played a role in the development of pulmonary hypertension. To demonstrate the effect of CXCR4 shRNA delivery on bone marrow cell migration, we investigated expression of GFP, a marker for donor bone marrow cells. We found a significant decrease in GFP expression in the lung from rats that had been transplanted with CXCR4 shRNA bone marrow cells. To further determine whether inhibition of CXCR4 in bone marrow cells impacted bone marrow-derived progenitor cell migration, we examined a hematopoietic progenitor marker, c-kit, in pulmonary artery isolated from rats. We found a significant decrease in c-kit expression in the pulmonary artery from rats that received the bone marrow cells electroporated with CXCR4 shRNA as compared with other hypoxic control groups, which indicated the delivery of CXCR4 shRNA in bone marrow cells also affected bone marrow-derived progenitor cell migration. Recently, Gambaryan et al. [27] reported that the effect of CXCR4 antagonist on hypoxia-induced pulmonary hypertension and vascular remodeling in mice was associated with a significantly decreased number of perivascular c-kit^+ ^hematopoietic progenitor cells. These data on c-kit expression together with the result from GFP expression demonstrated that CXCR4 knock down by shRNA decreased bone marrow-derived progenitor cell migration to the lungs under hypoxia.

Since a recent report has shown that inhibition of systemic CXCR4 through the delivery of AMD3100 could have had an effect on SDF-1 expression, we analyzed SDF-1 expression in the lung of rats that received AMD3100. We did not find significant change in SDF-1 expression in the animals that received AMD3100 in this study (data not shown).

Studies have shown that CXCR4 expression can alter bone marrow engraftment and that high expression of CXCR4 is required for engraftment [46-48]. In this study, we observed a decrease in WBC and in CD34 and CD45 expression in bone marrow cells, although the change was not significant. Interestingly, Monaco et al. [49] found that CXCR4 was not critical for engraftment of AML CD34^+ ^cells in NOD/SCID mice. They found that acute myeloid leukemia (AML) CD34^+ ^cells with virtually absent CXCR4 expression were able to engraft, but the cells with high expression of CXCR4 did not. They also found that anti-CXCR4 antibody failed to block the engraftment of AML cells onto NOD/SCID mice. In addition, a recent study showed that inhibition of CXCR4 by the antagonist AMD3100 improved donor hematopoietic cell engraftment in a mouse model [50]. The different results observed in separate laboratories suggest that CXCR4 is important, but may not be critical for regulating engraftment of bone marrow cells.

In conclusion, this study found that CXCR4 plays an important role in development of hypoxia-induced pulmonary hypertension and vascular remodeling. We also found that specific inhibition of the CXCR4 in bone marrow cells attenuated hypoxia-induced pulmonary hypertension and vascular remodeling. Our data demonstrated the importance of CXCR4 in the development of chronic hypoxic pulmonary hypertension and vascular remodeling in rats and demonstrated the role of CXCR4 in regulation of bone marrow cell migration in that process. This study suggests a novel therapeutic approach for pulmonary hypertension by inhibiting bone marrow cell recruitment.

## Competing interests

The authors declare that they have no competing interests.

## Authors' contributions

LY initiated and designed this study, performed experiments and wrote manuscript. CH revised manuscript. All authors read and approved the final manuscript.
